# Effects of manganese-excess on CO_2 _assimilation, ribulose-1,5-bisphosphate carboxylase/oxygenase, carbohydrates and photosynthetic electron transport of leaves, and antioxidant systems of leaves and roots in *Citrus grandis *seedlings

**DOI:** 10.1186/1471-2229-10-42

**Published:** 2010-03-07

**Authors:** Qing Li, Li-Song Chen, Huan-Xin Jiang, Ning Tang, Lin-Tong Yang, Zheng-He Lin, Yan Li, Gang-Hua Yang

**Affiliations:** 1 Institute of Horticultural Plant Physiology, Biochemistry and Molecular Biology, Fujian Agriculture and Forestry University, Fuzhou 350002, China; 2 College of Horticulture, Fujian Agriculture and Forestry University, Fuzhou 350002, China; 3 Fujian Key Laboratory for Plant Molecular and Cell Biology, Fujian Agriculture and Forestry University, Fuzhou 350002,China; 4 College of Life Science, Fujian Agriculture and Forestry University, Fuzhou 350002, China; 5 College of Resource and Environment, Fujian Agriculture and Forestry University, Fuzhou 350002, China

## Abstract

**Background:**

Very little is known about the effects of manganese (Mn)-excess on citrus photosynthesis and antioxidant systems. Seedlings of sour pummelo (*Citrus grandis*) were irrigated for 17 weeks with nutrient solution containing 2 μM (control) or 500 μM (excess) MnSO_4_. The objective of this study were to understand the mechanisms by which Mn-excess leads to a decrease in CO_2 _assimilation and to test the hypothesis that Mn-induced changes in antioxidant systems differ between roots and leaves.

**Results:**

Mn-excess decreased CO_2 _assimilation and stomatal conductance, increased intercellular CO_2 _concentration, but did not affect chlorophyll (Chl) level. Both initial and total ribulose-1,5-bisphosphate carboxylase/oxygenase (Rubisco) activity in Mn-excess leaves decreased to a lesser extent than CO_2 _assimilation. Contents of glucose, fructose, starch and total nonstructural carbohydrates did not differ between Mn-excess leaves and controls, while sucrose content was higher in the former. Chl a fluorescence (OJIP) transients from Mn-excess leaves showed increased O-step and decreased P-step, accompanied by positive L- and K-bands. Mn-excess decreased maximum quantum yield of primary photochemistry (F_v_/F_m_) and total performance index (PI_tot,abs_), but increased relative variable fluorescence at I-steps (V_I_) and energy dissipation. On a protein basis, Mn-excess leaves displayed higher activities of monodehydroascorbate reductase (MDAR), glutathione reductase (GR), superoxide dismutase (SOD), catalase (CAT) and guaiacol peroxidase (GPX) and contents of antioxidants, similar ascorbate peroxidase (APX) activities and lower dehydroascorbate reductase (DHAR) activities; while Mn-excess roots had similar or lower activities of antioxidant enzymes and contents of antioxidants. Mn-excess did not affect malondialdehyde (MDA) content of roots and leaves.

**Conclusions:**

Mn-excess impaired the whole photosynthetic electron transport chain from the donor side of photosystem II (PSII) up to the reduction of end acceptors of photosystem I (PSI), thus limiting the production of reducing equivalents, and hence the rate of CO_2 _assimilation. Both the energy dissipation and the antioxidant systems were enhanced in Mn-excess leaves, while the antioxidant systems in Mn-excess roots were not up-regulated, but still remained high activity. The antioxidant systems in Mn-excess roots and leaves provided sufficient protection to them against oxidative damage.

## Background

Manganese (Mn) is an essential micronutrient required for the normal growth of higher plants. Like other heavy metals, however, Mn may become toxic when present in excess [1]. Acid soils comprise up to 50% of the world's potentially arable lands. After aluminum (Al), Mn toxicity is probably the most important factor limiting plant productivity in acid soils [2].

Previous studies have shown that Mn-excess can inhibit CO_2 _assimilation in many plants including tobacco (*Nicotiana tabacum *L.) [3,4], wheat (*Triticum aestivum *L.) [5,6], cucumber (*Cucumis sativus *L.) [7], ricebean (*Vigna umbellata *L.) [8], white birch (*Betula platyphylla *Suk.) [9], rice (*Oryza sativa *L.) [10], common bean (*Phaseolus vulgaris *L.) [11], mungbean (*Vigna radiat*a L.) [12], *Alnus hirsuta *Turcz., *Betula ermanii *Charm., *Ulmus davidiana *Planch. and *Acer mono *Maxim. [13]. Suresh et al. [14] observed a decrease in stomatal conductance and transpiration rate with increasing Mn content in soybean [*Glycine max *(L.) Merr.] and concluded that Mn interfered with stomatal regulation. Unfortunately, no other parameters related to photosynthesis were presented in this paper, and it was not possible to determine whether decreased stomatal conductance was a primary effect of Mn toxicity or a result of serious leaf damage. Nable et al. [4] showed that the inhibition of photosynthesis in tobacco leaves was not a consequence of decreased stomatal conductance, because both intercellular CO_2 _concentration and rate of transpiration were not affected. Similar results have been obtained for wheat [5], ricebean [8], rice [10] and cucumber [7]. Macfie and Taylor [6] reported that the photosynthetic rate per unit chlorophyll (Chl) decreased in the sensitive wheat cultivar as Mn concentration in solution increased, indicating that Mn exerted its toxic effect on both Chl content and photosynthesis per unit Chl. Mn-induced decrease in photosynthetic rate through the decrease of Chl content has also been reported for common bean [11]. In contrast, Nable et al. [4] observed that the decline of photosynthesis in tobacco leaves preceded Chl degradation. Houtz et al. [3] concluded that the inhibitory effect of Mn toxicity on photosynthesis was due to Mn^2+ ^induced modification of ribulose-1,5-bisphosphate carboxylase/oxygenase (Rubisco, EC 4.1.1.39) kinetics. Kitao et al. [9] suggested that excess Mn in white birch leaves affected the activities of the CO_2 _reduction cycle rather than the potential efficiency of photochemistry (F_v_/F_m_), leading to an increase in Q_A _reduction state and thermal energy dissipation, and a decrease in photosystem II (PSII) quantum efficiency (quantum yield of PSII). Similar results have been found in *Alnus hirsuta *Turcz., *Betula ermanii *Charm., *Ulmus davidiana *Planch. and *Acer mono *Maxim. [13]. However, Chatterjee et al. [15] showed that *in vitro *Rubisco activity did not change in wheat plants treated with excess Mn, while Hill reaction activity was lower. The activities of photochemistry including Hill, photosystem I (PSI) and PSII partial reactions of chloroplasts from Mn-excess tobacco leaves were reported to remain constant despite ultimate development of severe necrosis [4], but Mn-excess decreased CO_2 _assimilation, F_v_/F_m _and PSII quantum efficiency in cucumber leaves [7]. Sinha et al. [12] showed that Mn toxicity decreased Hill activity of chloroplast isolated from mungbean leaves and photosynthetic rate in term of CO_2 _uptake. Doncheva et al. [16] reported that Mn-excess did not affect F_v_/F_m _and PSII quantum efficiency in Mn-tolerant maize (*Zea mays *L.) 'Kneja 434', but the two parameters significantly decreased in Mn-sensitive maize 'Kneja 605' at the highest Mn concentration. Experiment with wheat chloroplast suggested that the decrease in photosynthesis by excess leaf Mn was due to the peroxidative impairment of the thylakoid membrane function [17]. St. Clair et al. [18] observed that high Mn impaired the photosynthetic function of sugar maple (*Acer saccharum *Marsh.) and red maple (*Acer rubrum *L.), particularly in high light conditions, but antioxidant enzyme and quantum yield of PSII/quantum yield of CO_2 _fixation data suggested that this response was not the result of photo-oxidative stress. Therefore, the mechanisms by which Mn-excess leads to a decrease in CO_2 _assimilation are still not well understood.

Mn toxicity can induce oxidative stress through direct generation of reactive oxygen species (ROS) from Mn ions in the Fenton reaction [19] or direct transfer of electrons in single reaction, leading to a rise ROS level [20,21]. To minimize cellular damage caused by ROS, plants have evolved a scavenging system composed of antioxidants such as ascorbate (ASC) and reduced glutathione (GSH) and antioxidant enzymes such as superoxide dismutase (SOD, EC 1.15.1.1), ascorbate peroxidase (APX, EC 1.11.1.11), glutathione reductase (GR, EC 1.6.4.2), monodehydroascorbate reductase (MDAR, EC 1.6.5.4), and dehydroascorbate reductase (DHAR, EC 1.8.5.1), catalase (CAT, EC 1.11.1.16) and guaiacol peroxidase (GPX, EC 1.11.1.7) [22,23]. Despite the large body of evidence concerning the effects of Mn toxicity on the antioxidant systems in plant leaves [7,18,21,23-26], very little is known about the effects of Mn-excess on root antioxidant systems. Shi et al. [23] reported that in cucumber roots, Mn-excess increased the activities of Mn-SOD and Fe-SOD, but decreased the activities of Cu/Zn-SOD and CAT, and Mn-excess also affected the activities of GPX, APX, DHAR and GR. Experiments with other heavy metals have shown that the changes in antioxidant systems differ between roots and leaves in response to excess heavy metals [27-29], and may be a response to Mn-excess. In a study, Boojar and Goodariz [20] reported that the activities of SOD, CAT and GPX in the roots and leaves of *Datura stramonium*, and *Chenopodium ambrosioides *were enhanced in response to Mn-excess. Unfortunately, the activities of other antioxidant enzymes and the contents of ASC, dehydroascorbate (DHA), GSH and oxidized glutathione (GSSG) were not presented in this paper.

Citrus belongs to evergreen subtropical fruit trees and is cultivated in humid and subhumid of tropical, subtropical, and temperate regions of the world mainly on acid soils. Although the effects of Mn-excess on leaf structure and chloroplast ultrastructure of *Citrus volkameriana *L. have been investigated [30], there is hardly any information on photosynthesis and antioxidant systems of citrus in response to Mn-excess. In this study, we investigated the effects of Mn-excess on CO_2 _assimilation, Rubisco, carbohydrates and photosynthetic electron transport in leaves, and antioxidant systems in roots and leaves of sour pummelo [*Citrus grandis *(L.) Osbeck]. The objective of this study were to understand the mechanisms by which Mn-excess leads to a decrease in CO_2 _assimilation and to test the hypothesis that Mn-induced changes in antioxidant systems differ between roots and leaves.

## Results

### Seedling growth and specific weight

Mn-excess decreased root, stem and leaf dry weight (DW), and specific leaf weight. Leaf and stem DW decreased to a larger extent than root DW in response to Mn-excess, and resulted in a greater root DW/shoot DW ratio (Table 1).

**Table 1:** Effects of Mn-excess on leaf, stem and root DW, and specific leaf weight in sour pummelo seedlings

Treatments	Root DW (g plant^-1^)	Stem DW (g plant^-1^)	Leaf DW (g plant^-1^)	Root DW/Shoot DW	Specific leaf weight	
					
					(g FW m^-2^)	(g DW m^-2^)
Control	5.83 ± 0.68 a	6.16 ± 0.64 a	10.41 ± 0.59 a	0.35 ± 0.02 b	275 ± 4 a	124 ± 3 a
Mn-excess	3.68 ± 0.62 b	3.16 ± 0.68 b	5.78 ± 0.86 b	0.41 ± 0.02 a	244 ± 10 b	109 ± 5 b

Data are means ± standard errors (*n *= 5 or 10). Within a column, values followed by different letters are significantly different at *P *< 0.05.

### Mn, total soluble protein, Chl and carotenoids (Car)

Mn-excess increased root, stem and leaf Mn content, but decreased leaf soluble protein content expressed on a leaf area (Table 2), fresh weight (FW) or DW (data not shown) basis and root soluble protein expressed on a root FW (Table 2) or DW (data not shown) basis.

**Table 2:** Effects of Mn-excess on Mn content of roots, stems and leaves, and total soluble protein content of roots and leaves in sour pummelo seedlings

Treatments	Mn (μ g g^-1 ^DW)			Soluble protein	
		
	Roots	Stems	Leaves	Leaves (g m^-2^)	Roots (mg g^-1 ^FW)
Control	124.5 ± 11.7 b	6.5 ± 0.4 b	18.4 ± 1.8 b	8.4 ± 0.2 a	10.6 ± 0.5 a
Mn-excess	11471.2 ± 1457.8 a	583.6 ± 101.2 a	906.3 ± 123.1 a	6.5 ± 0.2 b	8.2 ± 0.5 b

Data are means ± standard errors (*n *= 5-8). Within a column, values followed by different letters are significantly different at *P *< 0.05.

Mn-excess did not significantly affect the contents of Chl, Chl a, Chl b and Car, whether the data were expressed on a leaf area (Table 3), FW or DW (data not shown) basis. Mn-excess significantly decreased the ratio of Chl a/b, but significantly increased the ratio of Car/Chl (Table 3).

**Table 3:** Effects of Mn-excess on Chl and Car contents in sour pummelo leaves

Treatments	Chl (mg m^-2^)	Chl a (mg m^-2^)	Chl b (mg m^-2^)	Chl a/b	Car (mg m^-2^)	Car/Chl
Control	587 ± 24 a	435 ± 16 a	152 ± 8 a	2.88 ± 0.04 a	118 ± 5 a	0.201 ± 0.003 b
Mn-excess	526 ± 28 a	384 ± 22 a	141 ± 5 a	2.72 ± 0.06 b	112 ± 5 a	0.213 ± 0.002 a

Data are means ± standard errors (*n *= 6). Within a column, values followed by different letters are significantly different at *P *< 0.05.

### Leaf gas exchange and Rubisco

**Figure 1:**
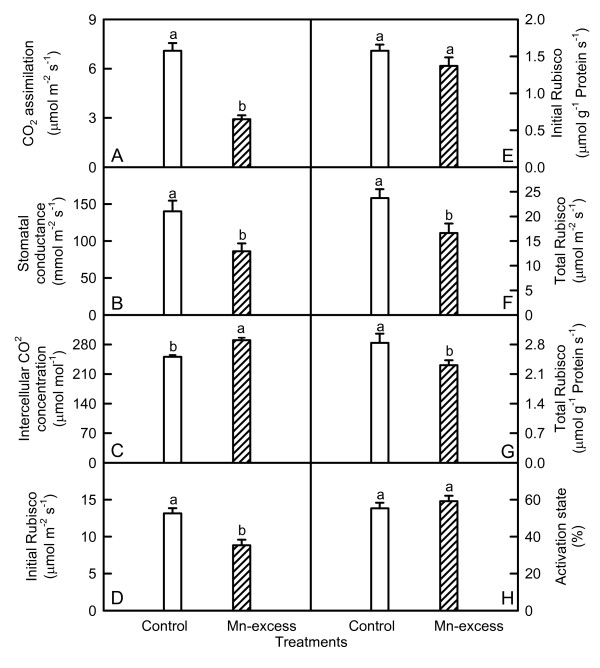
**Effects of Mn-excess on CO_2_assimilation (A), stomatal conductance (B), and intercellular CO_2 _concentration (C), initial Rubisco activity (D and E), total Rubisco activity (F and G) and Rubisco activation state (H) in sour pummelo leaves**. Bars represent means ± standard errors (*n *= 4-8). Different letters above standard error bars indicate significant differences at *P *< 0.05.

Both CO_2 _assimilation (Fig. 1A) and stomatal conductance (Fig. 1B) significantly decreased, but intercellular CO_2 _concentration (Fig. 1C) significantly increased in Mn-excess leaves compared with controls.

Both initial and total Rubisco activity was significantly lower in Mn-excess leaves than in controls except for a similar initial activity expressed on a leaf protein basis between the Mn treatments (Fig. 1D-G), while Mn-excess did not significantly affect Rubisco activation state (Fig. 1H).

### Leaf nonstructural carbohydrates

**Figure 2:**
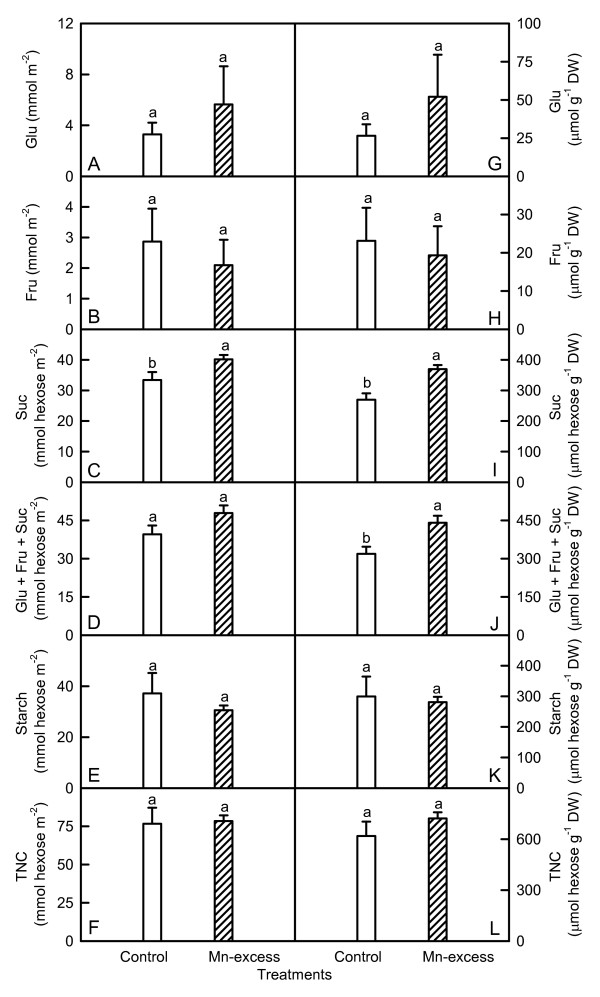
**Effects of Mn-excess on contents of glucose (Glu, A and G), fructose (Fru, B and H), sucrose (Suc, C and I), Glu + Fru + Suc (D and J), starch (E and K) and total nonstructural carbohydrates (TNC, F and L) of sour pummelo leaves expressed on a leaf area (A-F) or DW (G-L) basis**. Bars represent means ± standard errors (*n *= 6). Different letters above standard error bars indicate significant differences at *P *< 0.05.

As shown in Fig. 2, there were no significant differences in the contents of glucose, fructose, starch, and total nonstructural carbohydrates (TNC) between the Mn treatments regardless of how the data were expressed, while sucrose content was significantly higher in Mn-excess leaves. Expressed on a DW basis, Mn-excess leaves displayed a higher content of soluble sugars (glucose + fructose + sucrose), but a similar content of soluble sugars on an area basis.

### Leaf Chl a fluorescence (OJIP) transients and related parameters

**Figure 3:**
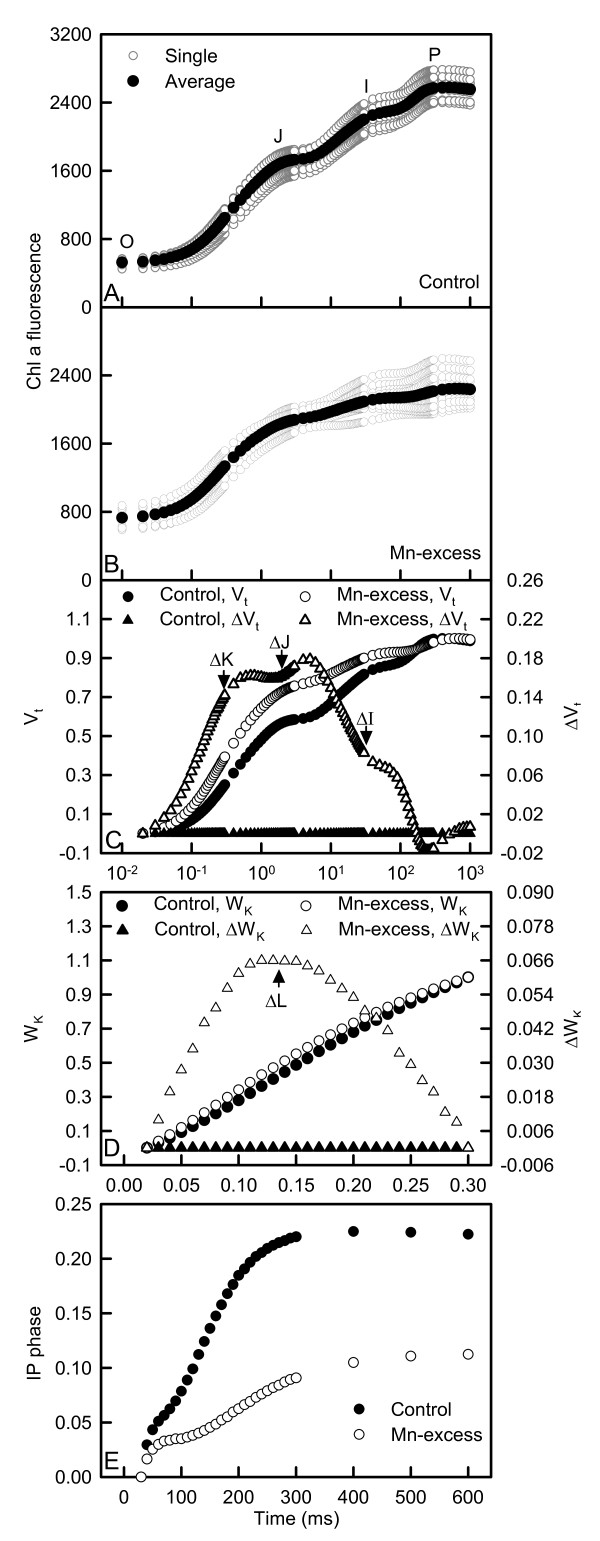
Effects of Mn-excess on OJIP transients (A and B), and the different expressions of relative variable fluorescence derived from the average OJIP transients: (C) between F_o _and F_m_: V_t_= (F_t _-- F_o_)/(F_m _-- F_o_) and the differences of the two samples to the control sample (ΔV_t_); (D) between F_o _and F_300 μ*s*_: W_K _= (F_t _-- F_o_)/(F_300 μs_-- F_o_) and the differences of the two samples to the control sample (ΔW_K_) and (E) IP phase: (F_t _-- F_o_)/(F_I _-- F_o_) -- 1 = (F_t _-- F_I_)/(F_I _-- F_o_) in dark-adapted sour pummelo leaves.

All OJIP transients from both Mn-excess and control leaves displayed a typical polyphasic rise with the basic steps of O-J-I-P. Mn-excess resulted in an increase in the heterogeneity of samples. OJIP transients from Mn-excess leaves showed a rise at the O-step and a depression at the P-step (Fig. 3A-B).

As shown in Fig. 3C-E, Mn-excess leaves displayed positive ΔL-, ΔK-, ΔJ- and ΔI-bands compared with controls around 130 μs, 300 μs, 2 ms and 30 ms, respectively and decreased the maximum amplitude of IP phase.

**Figure 4:**
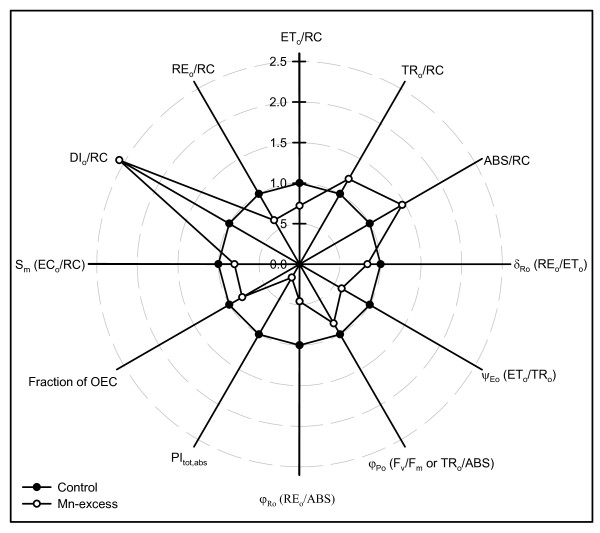
**Effects of Mn-excess on S_m_(EC_o_/RC), DI_o_/RC, RE_o_/RC, ET_o_/RC, TR_o_/RC, ABS/RC, δ_Ro _(RE_o_/ET_o_), ψ_Eo _(ET_o_/TR_o_), φ_Po _(F_v_/F_m _or TR_o_/ABS), φ_Ro _(RE_o_/ABS), PI_tot,abs _and fraction of OEC in dark-adapted sour pummelo leaves**. Each point is the mean of 9 or 10 replicates. All the values were expressed relative to the control (set as 1). All the parameters were significantly higher or lower in Mn-excess leaves than in controls.

Compared with controls, Mn-excess leaves had decreased total electron carriers per reaction center (S_m _or EC_o_/RC), reduction of end acceptors at PSI electron acceptor side per RC (RE_o_/RC), electron transport flux per RC (ET_o_/RC), efficiency with which an electron can move from the reduced intersystem electron acceptors to the PSI end electron acceptors (δ_Ro _or RE_o_/ET_o_), probability that a trapped exciton moves an electron into the electron transport chain beyond Q_A _^- ^(ψ_Eo _or ET_o_/TR_o_), maximum quantum yield of primary photochemistry (φ_Po _or F_v_/F_m _or TR_o_/ABS), quantum yield for the reduction of end acceptors of PSI per photon absorbed (φ_Ro _or RE_o_/ABS) and total performance index (PI_tot,abs_), but increased dissipated energy per RC (DI_o_/RC), trapped energy flux per RC (TR_o_/RC), absorption flux per RC (ABS/RC) and inactivation of oxygen evolving complex (OEC) (Fig. 4).

### Antioxidant enzymes and antioxidants, and malondialdehyde (MDA)

**Figure 5:**
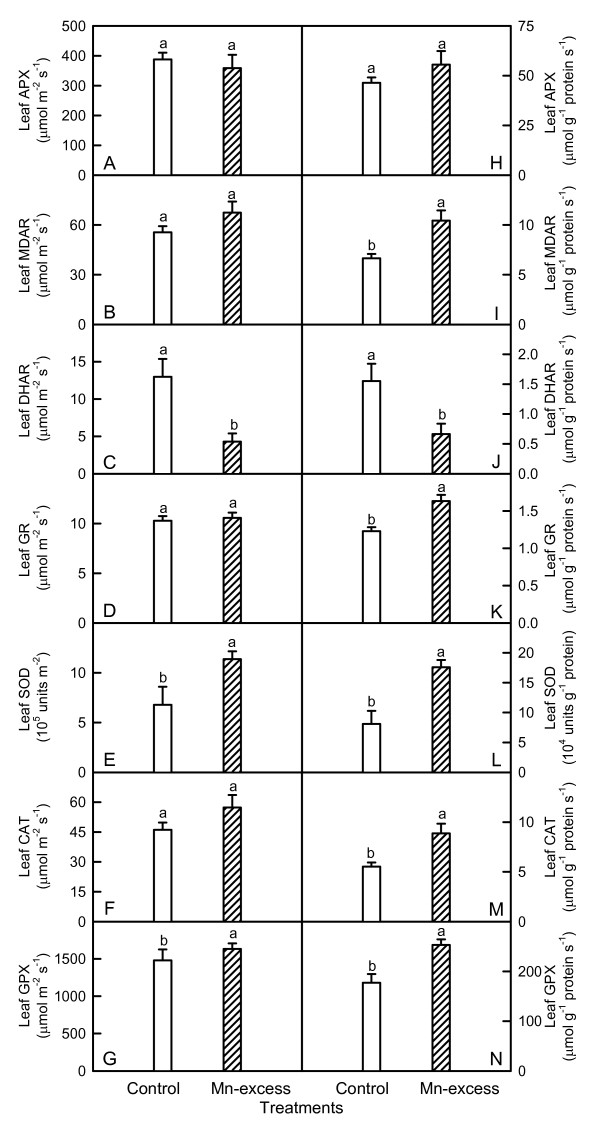
**Effects of Mn-excess on activities of APX (A and H), MDAR (B and I), DHAR (C and J), GR (D and K), SOD (E and L), CAT (F and M) and GPX (G and N) of sour pummelo leaves expressed on a leaf area (A-G) or protein (H-N) basis**. Bars represent means ± standard errors (*n *= 5-6). Different letters above standard error bars indicate significant differences at *P *< 0.05.

**Figure 6:**
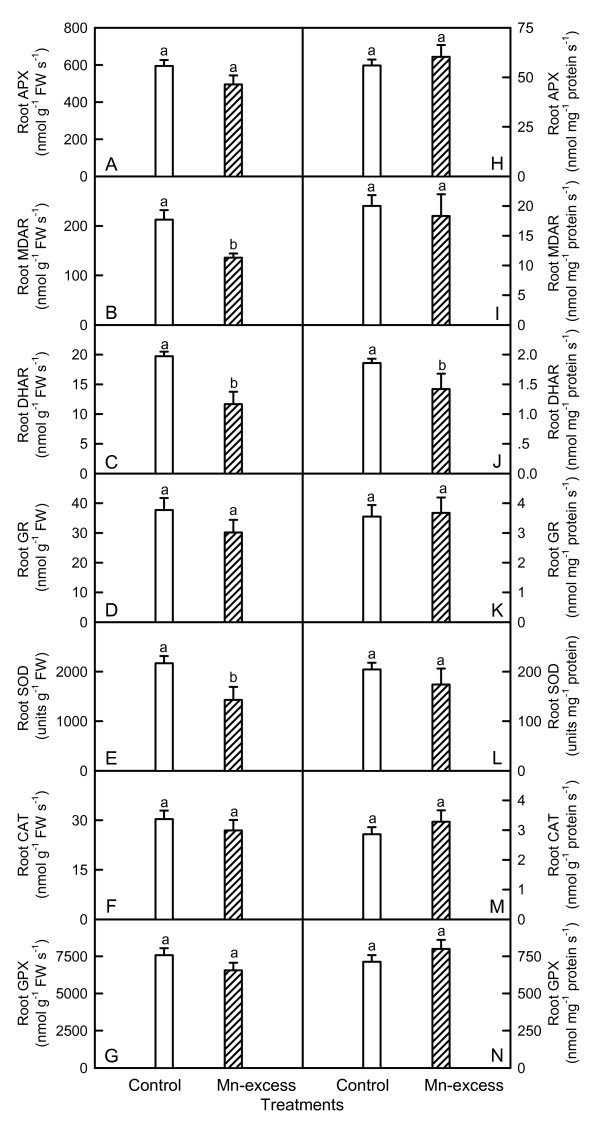
**Effects of Mn-excess on activities of APX (A and H), MDAR (B and I), DHAR (C and J), GR (D and K), SOD (E and L), CAT (F and M) and GPX (G and N) of sour pummelo roots expressed on a root FW (A-G) or protein (H-N) basis**. Bars represent means ± standard errors (*n *= 5-7). Different letters above standard error bars indicate significant differences at *P *< 0.05.

Mn-excess leaves had higher or similar APX, MDAR, GR, SOD, CAT and GPX activities whether the results were expressed on a leaf area or protein basis, while DHAR activity were lower (Fig. 5). Conversely, Mn-excess roots displayed similar or lower APX, MDAR, DHAR, GR, SOD, CAT and GPX activities, regardless of how the data were expressed (Fig. 6).

**Figure 7:**
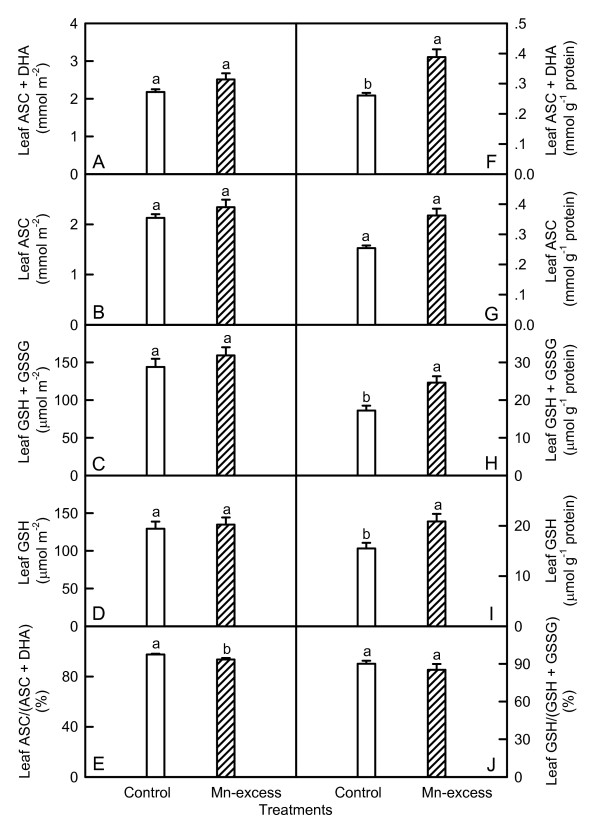
**Effects of Mn-excess on contents of ASC + DHA (A and F), ASC (B and G), GSH + GSSG (C and H), GSH (D and I) expressed on a leaf area (A-D) or protein (F-I) basis, and ratios of ASC to ASC + DHA (E) and GSH to GSH + GSSG (J) in sour pummelo leaves**. Bars represent means ± standard errors (*n *= 5-8). Different letters above standard error bars indicate significant differences at *P *< 0.05.

**Figure 8:**
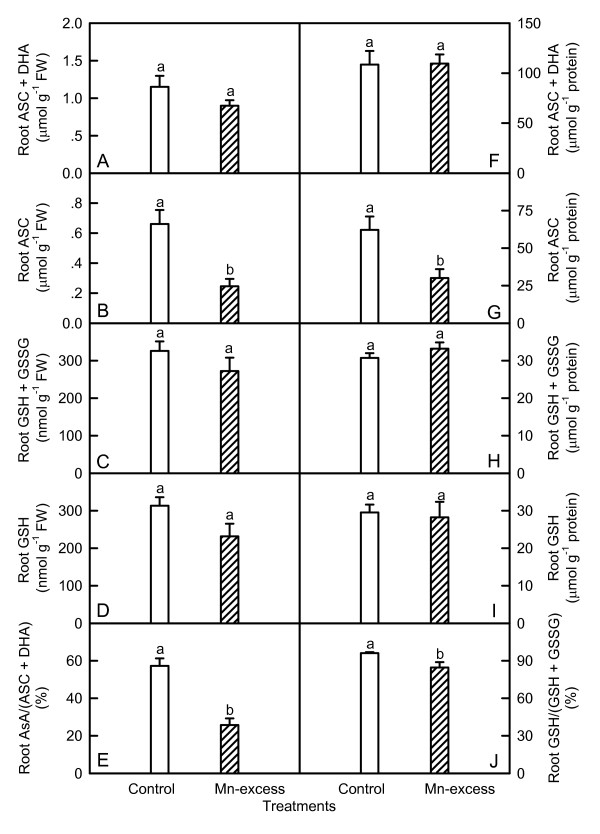
**Effects of Mn-excess on contents of ASC + DHA (A and F), ASC (B and G), GSH + GSSG (C and H), GSH (D and I) expressed on a root FW (A-D) or protein (F-I) basis, and ratios of ASC to ASC + DHA (E) and GSH to GSH + GSSG (J) in sour pummelo roots**. Bars represent means ± standard errors (*n *= 5-7). Different letters above standard error bars indicate significant differences at *P *< 0.05.

Mn-excess leaves showed similar contents of total ascorbate (ASC + DHA) (Fig. 7A), total glutathione (GSH + GSSG) (Fig. 7C) and GSH (Fig. 7D) on a leaf area basis and ASC on a leaf area or protein basis (Fig. 7B and 7G), but higher total ascorbate (Fig. 7F), total glutathione (Fig. 7H) and GSH (Fig. 7I) on a leaf protein basis. The ratio of GSH to total glutathione did not significantly differ between Mn-excess leaves and controls (Fig. 7J), while the ratio of ASC to total ascorbate was slightly lower in the former (Fig. 7E). There were no significant differences in the contents of total ascorbate (Fig. 8A and 8F), total glutathione (Fig. 8C and 8H) and GSH (Fig. 8D and 8I) between Mn-excess roots and controls, while the content of ASC (Fig. 8B and 8G) and the ratios of ASC/(ASC + DHA) (Fig. 8E) and GSH/(GSH + GSSG) (Fig. 8J) were significantly lower in Mn-excess roots than in controls.

**Figure 9:**
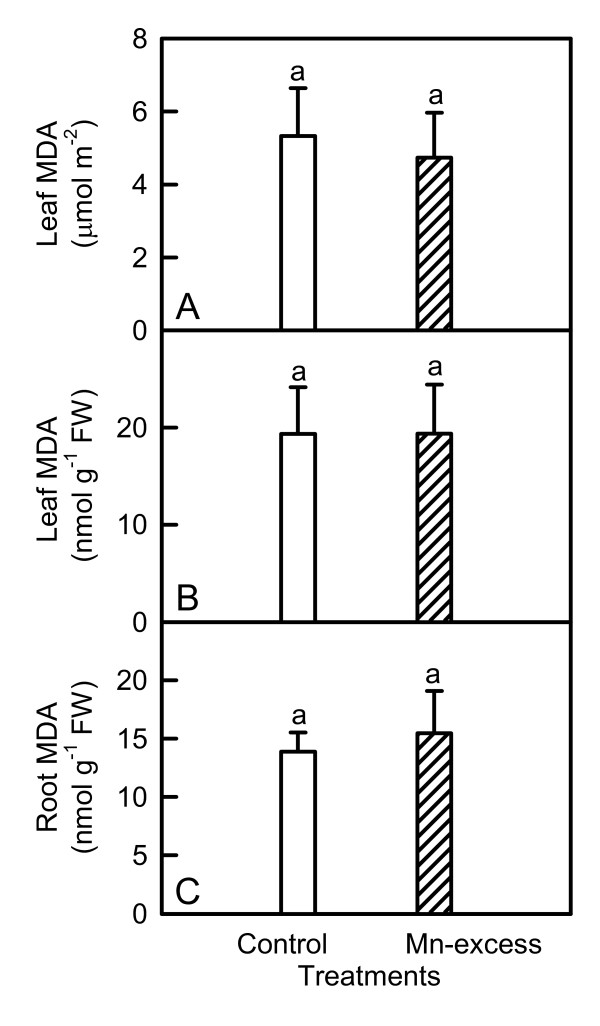
**Effects of Mn-excess on content of leaf MDA expressed on a leaf area (A) or FW (B) basis and of root MDA expressed on a FW basis (C) in sour pummelo seedlings**. Bars represent means ± standard errors (*n *= 5-6). Different letters above standard error bars indicate significant differences at *P *< 0.05.

As shown in Fig. 9, Mn-excess did not significantly affect MDA contents of roots and leaves.

## Discussion

The present work (Table 1), like that of previous workers [1,7,26,31] indicates that Mn-excess inhibits the plant growth. The higher ratio of root DW/shoot DW in Mn-excess plants (Table 1) agrees with the view that plant tops are affected by Mn toxicity to a greater extent than root systems [1]. However, Gherardi and Rengel [31] reported that Mn toxicity did not affect the ratio of root DW/shoot DW in lucerne (*Medicago sativa *L.).

The overwhelming majority of Mn was retained in the roots (Table 2), as previously found for lucerne [31]. According to Vose and Randall [32], tolerance to Mn toxicity is associated not only with low Mn uptake, but also with relatively little Mn translocation from roots to shoots. Mora et al. [33] reported that ryegrass (*Lolium perenn*e L.) cultivars tolerant to Mn-excess (Kingston and Jumbo) accumulated high Mn in roots and proportionally low Mn in shoots, while the sensitive ones (Aries and Nui) showed large Mn translocation from roots to shoots. Thus, the relatively low Mn content in upper parts of the Mn-excess plants (Table 2) might contribute to their tolerance for Mn-excess.

The lower CO_2 _assimilation in Mn-excess leaves (Fig. 1A) was primary caused by non-stomatal factors as the lower CO_2 _assimilation coincided with an increase in intercellular CO_2 _concentration (Fig. 1C). This agrees with the results obtained for wheat [5], tobacco [4], ricebean [8], rice [10] and cucumber [7]. However, Suresh et al. [14] concluded that Mn interfered with stomatal regulation.

Despite decreased CO_2 _assimilation (Fig. 1A), Mn-excess leaves had similar contents of nonstructural carbohydrates, except for a higher content of sucrose on a leaf area or DW basis and a higher content of soluble sugars on a DW basis (Fig. 2). This might result from the decreased demand for reduced C in growing sink tissues and less dilution due to growth inhibition (Table 1). However, Lidon [34] found that 2 mg L^-1 ^Mn treatment increased the content of starch, but decreased the content of soluble carbohydrates in rice shoots. In *Citrus volkameriana *L., the relative volume (%) of starch grains per chloroplast was 5-fold greater under 686 μM Mn than in the treatments with 0-98 μM Mn [30]. Evidence shows that soluble sugars, specifically hexoses, may repress photosynthetic gene expression, particularly of the nuclear-encoded small sub-unit of Rubisco, thus decreasing Rubisco content and CO_2 _assimilation [35]. Although Mn-excess leaves had slightly increased sucrose content (Fig. 2C and 2I), the contents of glucose (Fig. 2A and 2G) and fructose (Fig. 2B and 2H) did not significantly differ between the Mn treatments. This indicated that the feedback repression mechanism *via *accumulation of soluble sugars did not play a major role in determining the activity of Rubisco and the rate of CO_2 _assimilation in Mn-excess leaves. This inference was also supported by the results that both initial and total Rubisco activity in Mn-excess decreased to a lesser extent (Fig. 1D-G) than CO_2 _assimilation (Fig. 1A). Therefore, decreased CO_2 _assimilation in Mn-excess leaves could not be attributed to a decrease in Rubisco activity.

The decrease in leaf CO_2 _assimilation in response to Mn-excess could not be attributed to a decrease in Chl, because there were no significant differences in the contents of Chl, Chl a and Chl b between the Mn treatments (Table 3). Mn-excess led to a slight decrease in Chl a/b ratio (Table 3), as previously found for mungbean [12]. However, Chl a/b ratio in rice leaves did not show significant changes in response to Mn toxicity [10]. Aro et al. [36] reported that there was an inverse linear relationship between the sensitivity of pea (*Pisum sativum *L.) leaves to photoinhibition and Chl a/b ratio. Hence, Mn-excess leaves could be more susceptible to photoinhibition *in vivo *compared with normal ones.

The presence of a positive ΔL-band in Mn-excess leaves (Fig. 3D) agrees with the results obtained for Al-stressed [37] and B-stressed [38] sour pummelo, N-deficient cowpea [39] and P-deficient tea [*Camellia sinensis *(L.) O. Kuntze] [40]. According to the Grouping Concept [41] and JIP-test [39], the positive ΔL-band in Mn-excess leaves meant that the PSII units were less grouped or less energy was being exchanged between independent PSII units. Because the grouped conformation is more stable than the ungrouped one, the decreased grouping indicated that the PSII units of Mn-excess leaves had lost their stability and become more fragile. This was also supported by our data that Mn-excess increased the heterogeneity of the samples (Fig. 3A and 3B).

Our finding that Mn-excess leaves had a lower maximum quantum yield of primary photochemistry (φ_Po _or F_v_/F_m _or TR_o_/ABS) (Fig. 4) indicated that photoinhibitory damage to PSII complexes occurred in Mn-excess leaves [42,43]. Similar results have been obtained for cucumber [7] and Mn-sensitive maize [16]. However, Mn-induced decrease in CO_2 _assimilation was unaccompanied by decreased F_v_/F_m _in ricebean [8], white birch [9], rice [10], *Alnus hirsuta *Turcz., *Betula ermanii *Charm., *Ulmus davidiana *Planch. and *Acer mono *Maxim. [13]. The decrease in F_v_/F_m _was due to an increase in F_o _and a decrease in F_m _(Fig. 3A and 3B), as previously found for Al-treated [37] and B-deficient [38] sour pummelo and P-deficient tea [40]. The higher F_o _might be caused by the damage of OEC, because Mn-excess leaves had increased inactivation (Fig. 4), or it might relate to the accumulation of reduced Q_A _[44], because the physiological fractional reduction of Q_A _to Q_A _^-^, as indicated by the increase in approximated initial slope (in ms^-1^) of the fluorescence transient V = f(t) [M_o _= 4 (F_300 μs _- F_o_)/(F_m _- F_o_)] (Fig. 3C), increased in Mn-excess leaves. Quenching of F_m _in Mn-excess leaves might arise from the photoinhibitory quenching (qI), because an increase in F_o _with a quenched F_m _was observed in Mn-excess leaves (Fig. 3A and 3B) [45].

The Mn-induced positive ΔK-band in the OJIP transient is in agreement with the results obtained for Al-treated [37] and B-stressed [38] sour pummelo, and chromate (Cr)-treated *Lemna gibba *L. [46] and *Spirodela polyrhiza *(L.) Schleid. [47]. This indicated that the OEC was damaged and the energetic connectivity between photosynthetic units was changed [48]. This was also supported by the data that Mn-excess leaves had increased inactivation of OEC (Fig. 4) and less energy exchange between independent PSII units, as indicated by the positive ΔL-band (Fig. 3D). The fraction of electrons from the RCs at the acceptor side relates not only to the capacity of electron donation to the RCs, but also to the electron transport capacity from RCs to electron acceptors. The Mn-induced positive ΔI-band (Fig. 3C) indicated that the acceptor side of PSII in Mn-excess leaves was damaged more severely than the donor side of PSII, as previous suggest from *in vitro *studies that inactivation of the acceptor side might be the main mechanism underlying impairment of electron transport [36]. This was also supported by our data that Mn-excess leaves displayed a higher F_o _compared with controls (Fig. 3A and 3B). If the acceptor side of PSII is photoinhibited, the F_o _is significantly increased [49]. Relative variable fluorescence (V_I_) is a derived parameter and its increase can be due to an increase in F_I _or a decrease in F_m_, or both. Because Mn-excess leaves had lower F_I _and F_m _compared with controls (Fig. 3A and 3B), the increased V_I _in Mn-excess leaves might indicate a relative change in the proportion of Q_B_-non-reducing PSII RCs rather than an increase in the absolute amount of the Q_B_-non-reducing PSII RCs [37]. It has been suggested that the amplitude of IP phase is a measure of the amount of reduced end acceptors at PSI acceptor side and that the IP phase represents the last and slowest rate-limiting step of the photosynthetic electron transport chain [50]. Our results showed that Mn-excess largely decreased the maximum amplitude of IP phase (Fig. 3E). In addition, Mn-excess leaves had decreased S_m _(EC_o_/RC), RE_o_/RC, ET_o_/RC, δ_Ro _(RE_o_/ET_o_), ψ_Eo _(ET_o_/TR_o_) and φ_Ro _(RE_o_/ABS), and increased DI_o_/RC, TR_o_/RC and ABS/RC, and damaged all of the photochemical and non-photochemical redox reactions, as indicated by the decreases in the total performance index (PI_tot,abs_) (Fig. 4). Therefore, we concluded that Mn-excess impaired the whole photosynthetic electron transport chain from the donor side of PSII up to the reduction of end acceptors of PSI, thus limiting the production of reducing equivalents, and hence the rate of CO_2 _assimilation.

Since CO_2 _assimilation was decreased in Mn-excess leaves (Fig. 1A), only a fraction of the absorbed light energy was used in photosynthetic electron transport. Correspondingly, energy dissipation, as indicated by DI_o_/RC (Fig. 4), increased in Mn-excess leaves. The excess absorbed photon flux can also potentially lead to the production of ROS [51]. In addition, heavy metals have been demonstrated to stimulate formation of ROS in roots and leaves [7,52]. Thus, the production of ROS might be enhanced in Mn-excess roots and leaves. When the production of ROS is stimulated by stress, some protective antioxidant enzymes and antioxidants may be affected in plants. On a protein basis, Mn-excess leaves had higher or similar activities of antioxidant enzymes and contents of antioxidants, except for a lower activity of DHAR (Figs. 5H-N and 7F-I). The up-regulation of some antioxidant enzymes has also been found in the leaves of cucumber [7,25] and common bean [24] plants. In contrast to the leaf antioxidant enzymes and antioxidants, Mn-excess roots had similar or lower activities of antioxidant enzymes and contents of antioxidants on a protein basis (Figs. 6H-N and 8F-I). Our results clearly showed that on a protein basis, only DHAR activity in roots and leaves was decreased by excess Mn (Figs. 5H-N and 6H-N). Considering that the DHAR activity was the lowest among the enzymes in the ascorbate-glutathione cycle, it was likely that the DHAR-catalyzed reaction was not the main route for the regeneration. Previous studies showed that the ratios of ASC to ASC + DHA and GSH to GSH + GSSG decreased under oxidative stress [53-55]. In our study, the ratio of ASC to ASC + DHA was slightly lower in Mn-excess leaves than in controls (Fig. 7E), while Mn-excess did not significantly affect the ratio of GSH to GSH + GSSG (Fig. 7J). By contrast to leaves, the ratio of ASC to ASC + DHA was much lower in Mn-excess roots than in controls (Fig. 8E), while Mn-excess only slightly decreased the ratio of GSH to GSH + GSSG (Fig. 8J). Similar MDA content in roots and leaves between the Mn treatments (Fig. 9) indicated that the antioxidant systems in Mn-excess roots and leaves provided considerable protection to them against oxidative damage. Thus, the decrease in ASC/(ASC + DHA) ratio in Mn-excess roots and leaves (Figs. 7E and 8E) and GSH/(GSH + GSSG) ratio in Mn-excess roots (Fig. 8J) might indicate the equilibrium between utilization and regeneration of ASC and GSH was achieved at lower levels of ASC and GSH in the pool, and did not necessarily imply the Mn-excess roots and leaves were damaged by oxidative stress. Therefore, decreased CO_2 _assimilation in Mn-excess leaves could not be attributed to oxidative damage.

## Conclusions

Mn-excess impaired the whole photosynthetic electron transport chain from the donor side of PSII up to the reduction of end acceptors of PSI, thus limiting the production of reducing equivalents, and hence the rate of CO_2 _assimilation. Both the energy dissipation and the antioxidant systems were up-regulated in Mn-excess leaves, while the antioxidant systems in Mn-excess roots were not enhanced, but still remained high activity. The antioxidant systems in Mn-excess roots and leaves provided considerable protection to them against oxidative damage.

## Methods

### Plant culture and Mn treatments

This study was conducted outdoors from April to November, 2008 at Fujian Agriculture and Forestry University (FAFU). Seeds of sour pummelo [*Citrus grandis *(L.) Osbeck] were germinated in sand in plastic trays. Six weeks after germination, uniform seedlings with a single stem were selected and transplanted to 6 L pots containing sand. Seedlings, three to a pot, were grown outdoors at FAFU. Each pot was supplied with 500 mL of nutrient solution every two days. The nutrient solution contained the following macronutrients (in mM): KNO_3_, 1.25; Ca(NO_3_)_2_, 1; (NH_4_)H_2_PO_4_, 0.25; MgSO_4_, 0.5; micronutrients (in μM): H_3_BO_3_, 10; MnSO_4_, 2; ZnCl_2_, 2; CuSO_4_, 0.5; (NH_4_)_6_Mo_7_O_24_, 0.065; and Fe-EDTA, 20. Ten weeks after transplanting the treatment was applied for 17 weeks: until the end of the experiment, each pot was supplied every other day until dripping with nutrient solution (approx. 500 mL) containing 2 μM (control) or 500 μM (excess) MnSO_4_. At the end of the experiment, fully-expanded (about 7 weeks old) leaves from different replicates and treatments were used for all the measurements. Leaf discs (0.58 cm^2 ^in size) were collected at noon under full sun and immediately frozen in liquid nitrogen. Approximately 10-mm-long root apices were excised from the same seedlings used for sampling leaves and frozen immediately in liquid nitrogen. Both leaf and root samples were stored at -80°C until extraction.

### Measurements of root, stem and leaf DW, and specific leaf weight

At the end of the experiment, 10 plants per treatment from different pots were harvested. The plants were divided into their separate parts (roots, stems and leaves). The plant material was then dried at 80°C for 48 h and the DW measured. Specific leaf weight was measured according to Syvertsen et al. [56].

### Determination of pigments, total soluble protein, and Mn

Leaf Chl, Chl a, Chl b, and Car were assayed according to Lichtenthaler [57]. Briefly, 2 frozen leaf discs were extracted with 8 mL of 80% (v/v) acetone for 24 h in the dark. The extracts were determined using Libra S22 ultraviolet-visible spectrophotometer (Biochrom Ltd., Cambridge, UK). Root and leaf total soluble protein was extracted with 50 mM Na_2_HPO_4_-KH_2_PO_4 _(pH 7.0) and 5% (w/v) insoluble polyvinylpolypyrrolidone (PVPP), and determined according to Bradford [58] using bovine serum albumin (BSA) as standard. Mn content in roots, stems and leaves was determined by atomic absorption spectroscopy after digested with 1 N HCl.

### Leaf gas exchange measurements

Measurements were made with a CIARS-2 portable photosynthesis system (PP systems, Herts, UK) at ambient CO_2 _concentration under a controlled light intensity of 1000 μmol m^-2 ^s^-1 ^between 9:30 and 10:30 on a clear day. During measurements, leaf temperature and vapor pressure deficit (VPD) were 26.9 ± 1.1°C and 2.0 ± 0.1 kPa, respectively.

### Leaf Rubisco activity measurements

Rubisco was extracted according to Chen et al. [59]. Two frozen leaf discs from the same leaf were ground with a pre-cooled mortar and pestle in 1 mL of extraction buffer containing 50 mM Hepes-KOH (pH 7.5), 10 mM MgCl_2_, 2 mM ethylenediaminetetraacetic acid (EDTA), 10 mM dithiothreitol (DDT), 1% (v/v) Triton X-100, 5% (w/v) insoluble PVPP, 1% (w/v) BSA, 10% (v/v) glycerol. The extract was centrifuged at 13 000 g for 40 s in 2°C, and the supernatant was used immediately for the assay of Rubisco activity. Rubisco activity was determined according to Lin et al. [40]. For initial activity, 50 μL of sample extract was added to a cuvette containing 900 μL of assay solution, immediately followed by adding 50 μL of 10 mM ribulose-1,5-biphosphate (RuBP), then mixing well. The change of absorbance at 340 nm was monitored for 40 s. For total activity, 50 μL of 10 mM RuBP was added 15 min later, after 50 μL of sample extract was combined with 900 μL of assay solution to fully activate all the Rubisco. The assay solution for both initial and total activity measurements, whose final volume was 1 mL, contained 100 mM Hepes-KOH (pH 8.0), 25 mM KHCO_3_, 20 mM MgCl_2_, 3.5 mM ATP, 5 mM phosphocretaine, 5 units of NAD-glyceraldehyde-3-phosphate dehydrogenase (NAD-GAPDH, EC 1.2.1.12), 5 units of 3-phosphoglyceric phospokinase (PCK, EC 2.7.2.3), 17.5 units of creatine phosphokinase (EC 2.7.3.2), 0.25 mM NADH, 0.5 mM RuBP, and 50 μL of sample extract. Rubisco activation state was calculated as the ratio of initial activity to total activity.

### Assay of leaf nonstructural carbohydrates

Sucrose, fructose, glucose and starch were extracted and assayed according to Chen and Cheng [60].

### Measurements of leaf OJIP transients

OJIP transient was measured by a Handy Plant Efficiency Analyzer (Handy PEA, Hansatech Instruments Limited, Norfolk, UK) according to Strasser et al. [61]. All the measurements were done with 3 h dark-adapted plants at room temperature.

OJIP transient was analyzed according to the JIP test [37,38,62,63]. The following data from the original measurements were used: the fluorescence intensity at 20 μs (considered as minimum fluorescence F_o_); the maximal fluorescence intensity, F_P_, equal to F_m _since the excitation intensity was high enough to ensure the closure of all RCs of PSII; the fluorescence intensity at 300 μs (F_300_μs), 2 ms (J-step, F_J_) and 30 ms (I-step, F_I_). The JIP test represents a translation of the original data to biophysical parameters and the performance index. The following parameters that all refer to time 0 (start of fluorescence induction) are: (*a*) relative variable fluorescence at the J-step [V_J _= (F_J _- F_o_)/(F_m _- F_o_)] and at the I-step [V_I _= (F_I _- F_o_)/(F_m _- F_o_)]; (*b*) normalized total complementary area above the OJIP transient or total electron carriers per RC (S_m _= EC_o_/RC) and the fraction of OEC in comparison with control [(1-V_K_/V_J_)_treated sample_/(1-V_K_/V_J_)_control_, where V_K _is the relative variable fluorescence at 300 μs]; (*c*) the specific energy fluxes per RC for absorption (ABS/RC), trapping (TR_o_/RC), electron transport (ET_o_/RC), dissipation (DI_o_/RC) and reduction of end acceptors at PSI acceptor side (RE_o_/RC); (*d*) the flux ratios or yields, i.e. the maximum quantum yield of primary photochemistry (φ_Po _= TR_o_/ABS = F_v_/F_m_), the probability that a trapped exciton moves an electron into the electron transport chain beyond Q_A _^- ^(ψ_Eo _= ET_o_/TR_o_), the quantum yield for the reduction of end acceptors of PSI per photon absorbed (φ_Ro _= RE_o_/ABS) and the efficiency with which an electron can move from the reduced intersystem electron acceptors to the PSI end electron acceptors (δ_Ro _= RE_o_/ET_o_); (*e*) the total performance index (PI_tot,abs_), measuring the performance up to the PSI end electron acceptors (PI_tot,abs _= (RC/ABS) × (φ_Po_/(1- φ_Po_)) × (ψ_Eo_/(1 - ψ_Eo_)) × (δ_Ro_/(1 - δ_Ro_)); (*f*) the IP phase (IP phase = (F_t _- F_o_)/(F_I _- F_o_) - 1 = (F_t _- F_I_)/(F_I _- F_o_), where F_t _is the fluorescence intensity at time t after onset of actinic illumination.

Extended analysis of OJIP transients was done by calculation of the relative variable fluorescence [37,63]: (A) between F_o _and F_m _[V_t _= (F_t _- F_o_)/(F_m _- F_o_)] and (B) between F_o _and F_300 μs _[W_K _= (F_t _- F_o_)/(F_300 μs_- F_o_)] and the differences between the treated and the control samples. Clear bands are visible in these transients, where treatments rise above the control transient which is the reference line. Positive ΔL-, ΔK-, ΔJ- and ΔI-bands appear around 130 μs, 300 μs, 2 ms and 30 ms, respectively, and are associated with the ungrouping of PSII units [41], the uncoupling of OEC [64], the accumulation of Q_A _^- ^[39] and the increased proportion of Q_B_-non-reducing PSII RCs [65,66], respectively.

### Antioxidant enzymes, antioxidants and MDA in leaves and roots

GPX, SOD, APX, MDAR, DHAR, GR and CAT in roots and leaves were extracted according to Chen and Cheng [51]. GPX was assayed at 470 nm (extinction coefficient 25.2 mM^-1 ^cm^-1^) in 1 mL of reaction mixture containing 100 mM potassium phosphate buffer (pH 6.0), 16 mM guaiacol, 5 μL of 10% (v/v) H_2_O_2 _and the enzyme extract. The reaction was started by adding the enzyme extract [67]. SOD activity was assayed according to Giannopolitis and Rice [68]. APX, CAT, MDAR, DHAR and GR were measured according to Chen and Cheng [51].

Frozen leaf discs or roots were ground in ice-cold 5% (w/v) TCA [49]. GSH and GSSG in the extract were determined according to Griffith [69]. Frozen leaf discs or roots were ground in ice-cold 6% (v/v) HClO_4_. ASC and DHA in the extract were measured according to Chen and Cheng [51]. MDA was extracted with 80% (v/v) ethanol and determined according to Hodges et al. [70].

### Experimental design and statistical analysis

There were 20 pots seedlings per treatment in a completely randomized design. Experiments were performed with 4 -10 replicates (one plant from different pots per replicate). Results represented the means ± standard errors. Unpaired *t*-test was applied for comparison between two means at *P *< 0.05 level.

## Abbreviations

APX: ascorbate peroxidase; ASC: ascorbate; Car: carotenoids; CAT: catalase; Chl: chlorophyll; DHA: dehydroascorbate; DHAR: dehydroascorbate reductase; DW: dry weight; ET_o_/TR_o_: probability (at time 0) that a trapped exciton moves an electron into the electron transport chain beyond Q_A _^-^; F_o_: minimum fluorescence; FW: fresh weight; GPX: guaiacol peroxidase; GR: glutathione reductase; GSH: reduced glutathione; GSSG: oxidized glutathione; MDA: malondialdehyde; MDAR: monodehydroascorbate reductase; Mn: manganese; OJIP: Chl a fluorescence; PI_tot,abs_: total performance index; RC: reaction center; RE_o_/ABS: quantum yield of electron transport from Q_A _^- ^to the PSI end electron acceptors; RE_o_/ET_o_: efficiency with which an electron can move from the reduced intersystem electron acceptors to the PSI end electron acceptors; ROS: reactive oxygen species; Rubisco: ribulose-1,5-bisphosphate carboxylase/oxygenase; RuBP: ribulose- 1,5-bisphosphate; SOD: superoxide dismutase; TNC: total nonstructural carbohydrates; TR_o_/ABS or F_v_/F_m_: maximum quantum yield of primary photochemistry at t = 0; V_I_: relative variable fluorescence at the I-step; V_J_: relative variable fluorescence at the J-step.

## Authors' contributions

QL performed most of the experiments and wrote the manuscript. LSC designed and directed the study and revised the manuscript. HXJ, NT, LTY, ZHL and GHY helped in measuring Rubisco activity and OJIP transients. YL helped in assaying Mn content. All authors have read and approved the final manuscript.
